# MNS16A tandem repeat minisatellite of human telomerase gene: functional studies in colorectal, lung and prostate cancer

**DOI:** 10.18632/oncotarget.15884

**Published:** 2017-03-03

**Authors:** Philipp Hofer, Cornelia Zöchmeister, Christian Behm, Stefanie Brezina, Andreas Baierl, Angelina Doriguzzi, Vanita Vanas, Klaus Holzmann, Hedwig Sutterlüty-Fall, Andrea Gsur

**Affiliations:** ^1^ Medical University of Vienna, Institute of Cancer Research, A-1090 Vienna, Austria; ^2^ University of Vienna, Department of Statistics and Operations Research, A-1010 Vienna, Austria

**Keywords:** genetic variation, MNS16A, functional polymorphism, telomerase, TERT regulation

## Abstract

MNS16A, a functional polymorphic tandem repeat minisatellite, is located in the promoter region of an antisense transcript of the human telomerase reverse transcriptase gene. MNS16A promoter activity depends on the variable number of tandem repeats (VNTR) presenting varying numbers of transcription factor binding sites for GATA binding protein 1. Although MNS16A has been investigated in multiple cancer epidemiology studies with incongruent findings, functional data of only two VNTRs (VNTR-243 and VNTR-302) were available thus far, linking the shorter VNTR to higher promoter activity.

For the first time, we investigated promoter activity of all six VNTRs of MNS16A in cell lines of colorectal, lung and prostate cancer using Luciferase reporter assay. In all investigated cell lines shorter VNTRs showed higher promoter activity. While this anticipated indirect linear relationship was affirmed for colorectal cancer SW480 (*P* = 0.006), a piecewise linear regression model provided significantly better model fit in lung cancer A-427 (*P* = 6.9 × 10^−9^) and prostate cancer LNCaP (*P* = 0.039). *In silico* search for transcription factor binding sites in MNS16A core repeat element suggested a higher degree of complexity involving X-box binding protein 1, general transcription factor II–I, and glucocorticoid receptor alpha in addition to GATA binding protein 1.

Further functional studies in additional cancers are requested to extend our knowledge of MNS16A functionality uncovering potential cancer type-specific differences. Risk alleles may vary in different malignancies and their determination *in vitro* could be relevant for interpretation of genotype data.

## INTRODUCTION

Telomeres protect the ends of eukaryotic chromosomes from exposure to DNA damage response and impending genomic instability [1]. Telomere shortening occurs during each cell division (replicative ageing) and usually leads to senescence of somatic cells. Cancer cells, in contrast, are able to overcome senescence by maintaining telomere length and to attain the potential for unrestrained proliferation, a hallmark of cancer. Predominantly, stabilization of telomere length is mediated by reactivation of telomerase activity, detectable in over 90% of all malignant tumors [3]. However, a considerable number of cancers counteracts telomere attrition via alternative lengthening of telomeres [3, 4], a mechanism based on homologous recombination of telomeres (recently reviewed [5]). Telomerase reverse transcriptase (*TERT*) gene encodes the catalytic subunit of telomerase holoenzyme. Elongation of shortened telomeres is enabled by TERT activity in conjunction with the telomerase RNA component (TERC) acting as template for DNA synthesis, dyskerin (DKC1) protein that binds and stabilizes TERC, as well as several other telomerase associated proteins [6–8].

Influence of telomerase activity on different malignancies has been demonstrated by abundant evidence predestinating telomerase as target for therapeutic approaches [9]. Genetic variations in *TERT* and other genes involved in telomere biology as well as their regulatory elements can substantially impact cancer susceptibility [10]. Genetic variants have the potential to serve as biomarkers for personal risk profiling of patients with diverse cancer types [9]. However, the functional mechanisms underlying the associations with cancer remain to be clarified for most risk variants.

In 2003, Wang *et al*. [11] first identified a polymorphic tandem repeat minisatellite of the human telomerase gene, termed MNS16A in a pilot study of lung cancer. MNS16A is located on chromosome 5p15.33 downstream of *TERT* exon 16 in the putative promoter region of a *TERT* antisense RNA transcript. The 23 bp core tandem repeat sequence can be separated by a CAT trinucleotide presenting a transcription factor binding site (TFBS) for GATA binding protein 1 (GATA-1). The different numbers of CAT insertions correlate with VNTR length and suggested a possible functionality of this polymorphism. In the initial experiments, Wang *et al*. [11] investigated promoter activity of two VNTRs in one non-small cell lung cancer cell line. The shorter VNTR showed lower promoter activity, while the longer VNTR was associated with increased risk of lung cancer [11]. Since then, MNS16A genotypes of over 19,000 individuals have been genotyped in many cancer types [12–24], but no further experimental follow-up on MNS16A functionality was published to our best knowledge thus far. In addition to the four VNTRs reported by Wang *et al*. (VNTR-243, VNTR-274, VNTR-302, VNTR-333) [11], we identified two novel VNTRs of MNS16A (VNTR-212 and VNTR-364) in case control studies of colorectal cancer [12] and prostate cancer [13]. This prompted us to investigate promoter activity of all known six MNS16A VNTRs in cell lines of colorectal, lung, and prostate cancer.

## RESULTS

**Figure 1:**
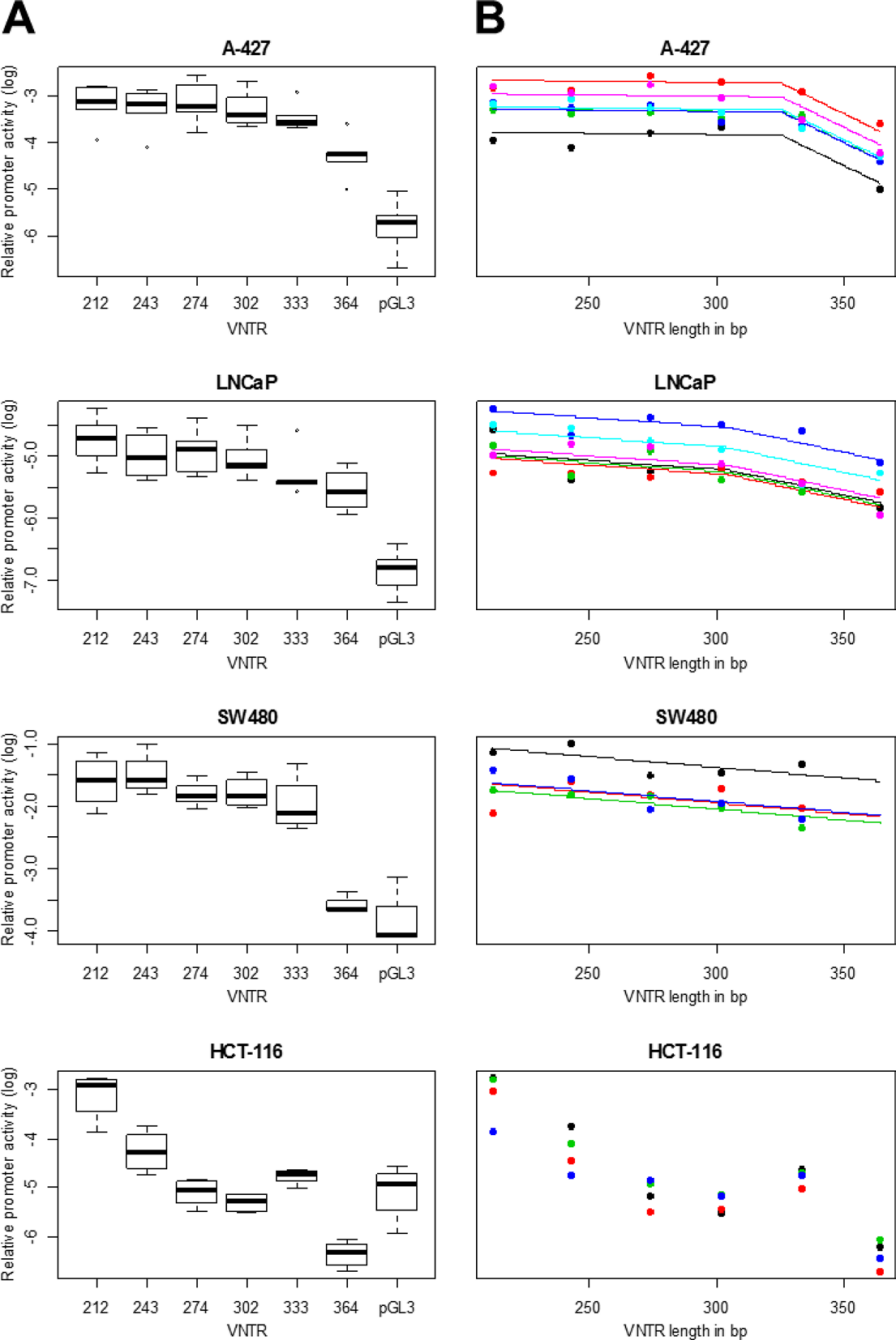
MNS16A promoter activity in different cancer cell lines (**A**) Relative promoter activities of different MNS16A VNTRs determined by Luciferase reporter assay are provided as Firefly/Renilla Luciferase ratios relative to CMV promoter positive control. Luminescence signals of untransfected cells (blank value) was subtracted and each Firefly/Renilla Luciferase ratio was divided by pGL3-CMV promoter activity. Log transformed values of relative promoter activities were plotted versus VNTR length. Each measurement was performed in duplicates and mean values of 4–6 experiments (A-427: *n* = 5, LNCaP: *n* = 6, SW480: *n* = 4, HCT-116: *n* = 4) are shown. (**B)** Each graph line represents an independent experiment. Regression models for each cell line were selected based on maximum regression coefficient R^2^. The piecewise model provided the best fit for cell lines A-427 and LNCaP. In SW480, VNTR-364 showed no significantly different promoter activity when compared to pGL3 negative control and was excluded from the model calculation. For HCT-116, no model was estimated because only VNTR-212 and −243 showed significant promoter activity.

Distribution of relative promoter activities of different MNS16A VNTRs determined by Luciferase reporter assays for different cell lines are provided in Figure 1A. In all investigated cell lines, promoter activity of shorter constructs (VNTRs) was higher than promoter activity of longer constructs reflecting an indirect correlation of VNTR length and promoter activity. In A-427 and LNCaP all VNTRs had significantly higher promoter activity when compared to pGL3 negative control. *P*-values of comparisons of pGL3 with VNTRs-212, −243, −274, −302, −333, −364 were 7.0 × 10^−5^, 9.0 × 10^−5^, 6.3 × 10^−5^, 3.5 × 10^−5^, 1.5 × 10^−4^, 1.5 × 10^−3^ (A-427) and 8.2 × 10^−5^, 3.3 × 10^−4^, 3.5 × 10^−4^, 9.1 × 10^−5^, 3.4 × 10^−4^, 3.2 × 10^−3^(LNCaP), respectively. For SW480 *P*-values were 8.7 × 10^−4^, 1.3 × 10^−4^, 8.1 × 10^−4^, 6.8 × 10^−4^, 1.2 × 10^−4^, 0.22, indicating, that VNTR-364 had no significant promoter activity. Only VNTRs with significant promoter activity were included in statistical models.

We modeled the shape of the relationship between construct length and promoter activity for each cell line by linear, and linear piecewise regression (Table 1). For the linear model, we observe a highly significant negative trend for all cell lines with *P*-values of 0.006 for SW480, 1.9 × 10^−7^ for LNCaP and 3.4 × 10^−6^ for A-427. For A-427 and LNCaP linear piecewise regression improved the model fit with significantly higher R^2^-values compared to simple linear regression (*P* = 6.9 × 10^−9^ for A-427 and *P* = 0.039 for LNCaP). Estimated theoretical breakpoints occurred at 326bp for A-427 (corresponding to VNTR-333) and at 304bp for LNCaP (corresponding to wild-type VNTR-302). Slopes to the right of the breakpoint were steeper than slopes to the left of the breakpoint for both cell lines (see also Figure 1B). This indicates a steeper decrease in relative promoter activity for larger VNTR lengths.

**Table 1:** Model coefficients of linear and linear piecewise regression models

	A-427		LNCaP		SW480	
**Linear regression**						
R^2^ linear trend	0.716		0.782		0.743	
Linear slope	−0.00589		−0.00492		−0.00340	
*P*-value linear slope	**3.4 × 10^−6^**	^**^*	**1.9 × 10^−7^**	^**^*	**0.00599**	^*^
**Piecewise regression**						
R^2^ piecewise	0.916		0.814		0.773	
*P*-value linear vs. piecewise	**6.9 × 10^−9^**	^**^*	**0.0389**	*	0.199	
Estimated breakpoint	325.7		304.0		223.0	
Slope left	−0.00066		−0.00285		0.01456	
Slope right	−0.02656		−0.00879		−0.00482	
*P*-value slope left	0.452		0.0216	*	0.294	
*P*-value slope right	**5.7 × 10^−11^**	^**^*	**8.6 × 10^−5^**	^**^*	**0.00567**	^*^

*P*-values < 0.05 were considered significant and are given in bold. Explained Variation (R^2^) of the selected models providing best fit are indicated by underline. Replicate was included as categorical variable in all models.

Regarding SW480, VNTR-364 showed no significant promoter activity and therefore was excluded from the models. Although R^2^ of the piecewise model (0.773) for SW480 was somewhat higher than R^2^ of the simple linear model (0.743), the difference was not significant (*P* = 0.199) and the linear model was selected providing the best model fit.

In order to study promoter activity of VNTR-364 in an additional colorectal cancer cell line, we also transfected HCT-116 and performed Luciferase assays. However, due to lower promoter activity than observed in SW480, we could not demonstrate promoter activity of all VNTRs in HCT-116. *P*-values of comparisons of pGL3 with VNTRs-212, −243, −274, −302, −333, −364 were 1.3 × 10^−4^, 0.024, 0.98, 0.56, 0.36, 0.017 (HCT-116). Although Luciferase data of independent experiments of different days yielded reproducible and comparable results for this cell line, solely the short VNTRs-212 and −243 exhibited significantly elevated promoter activity. VNTRs-274, −302 and −364 had luminescence levels below pGL3 negative control. This impeded estimation of a meaningful model for this cell line.

### Transcription factor binding sites

Search for TFBSs in MNS16A tandem repeats core sequence yielded five potential binding sites for four different transcription factors. In addition to GATA-1 (T00306), one TFBS for the general transcription factor II-I (TFII-I or GTF2I, T00824) and one for glucocorticoid receptor alpha (GR-alpha, T00337), as well as two sites for X-box binding protein 1 (XBP-1, T00902) were identified.

## DISCUSSION

Genotypes of MNS16A, a functional polymorphic tandem repeat minisatellite of *TERT* locus, have been investigated in cancers of the brain [14–16], breast [17–19], lung [11, 20, 21], colorectum [12], nasopharynx [22], prostate [13], blood [23], and kidney [24]. Furthermore, two meta-analyses of MNS16A were published so far [25, 26]. Both found significant overall associations with increased cancer risk. Stratification for cancer type showed pronounced associations for cerebral [25, 26] and breast cancer [26], but no significant elevation of lung cancer risk [25, 26]. Mode of analysis and overall findings of mostly well-designed and well-powered case-control studies were inconsistent. Thus, risk alleles could not be confirmed for different cancer types by independent studies. Increased risk of lung cancer was associated with longer alleles in an US hospital-based pilot scale study [11] but with the short VNTR-243 in a Korean study [21]. In our Austrian prostate cancer study [13], VNTR-274 was ascribed a protective effect in Caucasian males older than 70 years in stratified analysis, but no overall association with disease risk was observed. Furthermore, we investigated MNS16A genotypes in a Caucasian colorectal cancer cohort, reporting VNTR-274 as risk allele [12].

The mechanistic model behind MNS16A functionality bases on the observation, that VNTR-243, carrying less GATA-1 TFBSs than VNTR-302, was experimentally linked to increased promoter activity *in vitro* [11]. Increased formation of *TERT* antisense transcript may contribute to repression of *TERT* transcription and telomerase activity. Accordingly, Zhang *et al*. [22] observed that carriers of the SL genotype had lower TERT expression compared to LL carriers when analyzing nasopharyngeal carcinoma tissue by immunohistochemical staining. Repression of telomerase activity is tightly regulated in somatic human cells and a disturbance of this system can promote telomerase reactivation, a hallmark of carcinogenesis. To follow up on the initial findings of Wang *et al*. [11] we established clonal vectors of all known six VNTRs of MNS16A and investigated their promoter activity in colorectal, lung and prostate cancer cell lines.

Previously, we argued [12] that use of different classification systems categorizing MNS16A VNTRs into shorter (S), medium (M) and longer (L) alleles complicates inter-studies comparability and conduct of valuable meta-analyses [26]. Our present results emphasize that classification of VNTRs should be as exact as possible to reflect the total amount of repeat elements and TFBSs.

In all investigated cell lines we observed the anticipated indirect correlation between number of repeat elements and promoter activity. Although all cell lines exhibited a similar overall trend, model selection suggested higher complexity in A-427 and LNCaP. Especially in the lung cancer cell line A-427, the piecewise regression yielded significantly better model fit. VNTRs-212, −243, −274, −302, and −333 showed comparable promoter activity, while only for VNTR-364 a significant decrease was observed. In the prostate cancer cell line LNCaP, the piecewise model provided only a minute but significant improvement compared to the simple linear trend. In the colorectal cancer cell line SW480, promoter activity of VNTR-364 was too low and had to be excluded from statistical modeling yielding the linear model. Establishment of promoter activities of all VNTRs in colorectal cancer HCT-116 failed due to the low overall promoter activity in this cell line.

VNTRs with similar promoter activity could be assigned to the same functional subgroup. Translating this idea to interpretation of genotyping studies might be relevant if the identity of risk alleles was dependent on tissue or tumor type.

A strength of this study is that all six VNTRs were investigated simultaneously in cell lines of three common cancers for the first time. Thus far, solely GATA-1 was considered as relevant transcription factor behind MNS16A promoter activity. However, MNS16A tandem repeat core sequence presents also TFBSs for TFII-I, XBP-1, and GR-alpha. These transcription factors may be involved in MNS16A functionality as well. Occurrence and expression data of these transcription factors in different tissue and cancer types can be obtained from “The Human Protein Atlas” (HPA, Human Protein Atlas available fromwww.proteinatlas.org; accessed 22.Jul.2016) [27]. In summary, HPA protein data suggest distinct patterns of the four transcription factors potentially relevant for MNS16A functionality across different tissue and cancer types. Consideration of additional transcription factors, other than GATA-1, and their putative influence on MNS16A promoter activity adds further degrees of freedom to the numerous mechanisms of telomerase regulation at various levels. We hypothesize that MNS16A promoter activity is not only dependent of VNTR length, but also likely to be influenced by tumor type and the according risk allele, or set of risk alleles, may even be tissue-specific. Comparatively, mutations within the *TERT* promoter were recently recognized to induce activation of telomerase activity in multiple cancers [28, 29]. These genetic aberrations are one of the most frequent mutations in many cancers and are cancer type-specific in regard of their prevalence [30]. Activation of *TERT* promoter is thereby mediated by recruitment of transcription factors.

Presently, we confront an unexplored gap between association of cancer risk with MNS16A genotype determined from peripheral blood leukocytes and limited results from functional analysis in different tumor cell lines serving as experimental surrogates for different tumor types. Tissue-specific differences in MNS16A promoter activity could be causal for the differences reported by diverse genotyping studies. A tumor type-specific classification system for risk alleles may improve the interpretation of genotyping results generated by cancer epidemiology studies. Further studies are warranted not exclusively investigating germline genetic variation of the locus, but also functionality of MNS16A in consideration of tumor type and relevant regulatory factors.

## MATERIALS AND METHODS

### Construction of pGL-3_VNTR plasmids

For cloning of size-selected MNS16A PCR products [12, 13], TOPO TA Cloning Kit (Invitrogen, Carlsbad, CA, USA) containing pCRII-TOPO vector was used as described by the manufacturer. MNS16A VNTRs (−212, −243, −274, −302, −333, −364), excised from pCRII-TOPO were inserted into pGL3-basic vector (Promega, Madison, WI, USA) using Rapid DNA Ligation Kit (Roche Applied Science, Upper Bavaria, Germany). VNTR-212 was excised using restrictions enzymes (all Roche Applied Science) EcoR V and Sac I, VNTRs-243, −274, −302, −333 by using Xho I and Sac I, and VNTR-364 by use of Xho I and Hind III. pGL3-basic vector was digested using three combinations of restriction enzymes (Xho I and Hind III, Xho I and Sac I, SmaI and Sac I). Purification of vector DNA was performed by cesium chloride density gradient centrifugation (as recently described [31]). Verification of plasmids was performed by restriction enzyme digest.

### Tissue culture

All used cell lines were obtained from the American Type Culture Collection (Rockville, MD, USA): A-427 (lung adenocarcinoma), LNCaP (prostate carcinoma, derived from a metastatic lymph node), SW480 (Dukes B colorectal adenocarcinoma) and HCT-116 (colorectal carcinoma). Cells were cultured in Dulbecco's modified Eagle's medium supplemented with 10% fetal calf serum, 100 IU/mL penicillin and 100 μg/mL streptomycin at 37°C in presence of 7.5% carbon dioxide.

### Luciferase assay

Promoter activity of different pGL-3_VNTR constructs was measured in cells after transient calcium phosphate transfection using Dual-Luciferase Reporter Assay System (Promega) [31]. This assay depends on co-transfection of two different Luciferases. Firefly Luciferase served as experimental reporter and Renilla Luciferase as internal control. For A-427, LNCaP, and SW480 10 μg pGL-3_VNTR DNA and 3 μg pRL-SV40 DNA were co-transfected, while for HCT-116 8.5 μg pGL-3_VNTR and 1 μg Renilla pRL-SV40 DNA were used. Firefly luminescence signals were normalized to Renilla Luciferase. pGL-3-CMV-Luciferase (positive control) and pGL-3 basic vector (negative control) were included in all experiments. Between 150,000 and 300,000 cells were seeded and incubated 24 h upon transfection. All cell lysates were measured in duplicates three days after transfection. Final results were calculated from four to six independent experiments per cell line.

### Statistical analysis

To obtain relative promoter activities, luminescence signals of untransfected cells (blank value) were subtracted and Firefly/Renilla Luciferase ratio was divided by pGL3-CMV promoter activity. Descriptive analysis included assessment of relative promoter activities of different pGL-3_VNTR constructs for A-427, LNCaP, SW480, and HCT-116 cell lines, respectively, by calculating means and standard deviations. All further analyses were based on log relative promoter activity values. Log transformations lead to more symmetric distributions of promoter activities and more homogeneous variation within cell line and VNTR length. Box plots were used to illustrate distributions of log relative promoter activity for each cell line and VNTR length separately. Paired *t*-tests were performed to compare promoter activity of VNTRs with negative controls.

Relationships between MNS16A VNTR length and log promoter activity for A-427, LNCaP and SW480 were assessed by regression analysis with VNTR length as predictor for log promoter activity. Based on graphical analysis that suggested a change in the relationship at a certain VNTR length depending on cell line, two different regression models were considered: a model with a single linear term for VNTR length and a piecewise regression model with a change in the linear relationship at an optimal breakpoint that was estimated as part of the regression analysis. Model fits were assessed by diagnostic plots, R^2^-values and *P*-values for all regression coefficients.

As described, between four and six replicates (independent measurements) per cell line were performed. Promoter activity for all VNTR lengths was measured for each replicate. In order to account for the experimental design, an identifier for replicate was included as categorical variable in all regression models. *P*-values smaller than 5% were considered statistically significant. All statistical analyses were performed using *R* [32].

### Transcription factor binding motifs

PROMO version 3.0.2 [33, 34] (http://alggen.lsi.upc.es/cgi-bin/promo_v3/promo/promoinit.cgi?dirDB=TF_8.3; using TRANSFAC version 8.3; accessed 08.Jul.2016) was searched against MNS16A tandem repeat core sequence including CAT trinucleotide insertion (TCCTCTTAT catCTCCCAGTCTCATC). Only human factors and sites were considered and a dissimilarity rate of less than or equal to 10% was selected to search for potential TFBS within the submitted target sequence.
